# The effect of dietary omega-6 fatty acid enrichment in rodent models of military-relevant acute traumatic psychological stress and traumatic brain injury

**DOI:** 10.3389/frmbi.2024.1430340

**Published:** 2024-09-11

**Authors:** Matthew R. Rusling, James C. DeMar, Nabarun Chakraborty, Allison V. Hoke, Stacy Ann Miller, John G. Rosenberger, Andrew B. Batuure, Donna M. Wilder, Venkatasivasai Sujith Sajja, Joseph B. Long, Rasha Hammamieh, Aarti Gautam

**Affiliations:** ^1^ Medical Readiness Systems Biology Branch, Center for Military Psychiatry and Neuroscience, Walter Reed Army Institute of Research (WRAIR), Silver Spring, MD, United States; ^2^ Blast-Induced Neurotrauma Branch, Center for Military Psychiatry and Neuroscience, Walter Reed Army Institute of Research (WRAIR), Silver Spring, MD, United States

**Keywords:** omega-3, omega-6, microbiome, traumatic brain injury, post-traumatic stress disorder, linoleic acid, alpha-linolenic acid, rat

## Abstract

**Introduction:**

Sequelae from traumatic brain injuries (TBIs) and post-traumatic stress disorder (PTSD) are major career-limiting factors for combat soldiers. Overlap between TBI and PTSD symptoms alongside other common comorbidities complicate the diagnosis and treatment. Systems-level and high-throughput approaches are key in understanding the underlying biomolecular mechanisms and differentiating these conditions.

**Methods:**

The present study identifies dietary factors and proposes mechanisms behind psychological stress and TBI, using established preclinical animal models and a multi-omics approach. Here, we used microbiome characterizations of rats exposed to simulations of blast-induced TBI and underwater trauma (UWT)-induced psychological stress. We further studied the effect of dietary omega-6 versus omega-3 polyunsaturated fatty acid (n-6, n-3 PUFA) enrichment on the insult responses. The use of excess n-6 PUFA was chosen due to its high prevalence in the Western diet and pro-inflammatory nature. Prior to TBI or UWT, animals were maintained for 6 weeks and continued thereafter on either a standard diet or two customized chows imbalanced and diminished in omega-3 content. Corresponding shams were carried out for all groups. Fecal bacterial microbiome populations were assessed using 16S rRNA gene sequencing.

**Results:**

Physiologic outcome modeling identified that dietary status affected post-TBI lactate dehydrogenase (LDH) and triglyceride levels, with n-3 PUFA having a large attenuating influence. The UWT model showed similar trends, with diet significantly altering LDH, terminal corticosterone (14 days post-exposure), and a fear behavior susceptibility. Fecal microbiome alpha diversity was significantly reduced by high levels of n-3 PUFA. Likewise, beta diversity of the microbiome was significantly affected by both diet and time but not exposure to TBI or UWT. Compositionally, temporal effects on the microbiome were more likely to be observed with the diets. The most affected features fell within the *Proteobacteria* phyla, in which n-3 PUFA enrichment significantly reduced *Alphaproteobacteria* in the TBI model and increased *Gammaproteobacteria* in the UWT group.

**Discussion:**

All these observations can influence the vulnerability or resilience of the warfighter to blast-induced TBI and acute psychological stress. The microbiome mechanisms facilitate and provide a knowledge-driven unbiased panel of signatures to discriminate between the two insults and is an essential tool for designing precise care management.

## 1 Introduction

During Operations Iraqi Freedom and Enduring Freedom, there were significant increases in hospitalizations among service members due to traumatic brain injury (TBI) as well as mental disorders such as post-traumatic stress disorder (PTSD) and major depressive disorder (Staff, 2018). Many of these disorders coexist (Bryant, 2011; Weckle, 2013) because brain injuries are frequently sustained during psychologically traumatic situations, especially those faced in combat operations. There is an overlap between symptoms accompanying each condition, and the range of other comorbid problems that commonly coexist in the two conditions further complicates the diagnosis. Discrimination between psychological stress and TBI using a knowledge-driven unbiased panel of biomarker signatures would be extremely valuable in designing precise health care managements.

TBI involves damage to the brain from an external force, e.g., skull impact concussions, and the severity of TBI is typically described in terms of mild, moderate, or severe. the major source of closed-head TBI to soldiers in combat operations is blast exposure from bomb explosions, which results from three basic types of strikes to the skull and propagation of the kinetic energy forces to the brain: primary from overpressure waves, e.g., rapid air expansion; secondary from projectiles, e.g., thrown rocks; and tertiary from body displacement, e.g., falls against hard objects. These events mainly cause diffusive axonal shearing and compressive contusions to the brain’s neuronal cell layers, with accompanying tearing of blood vessels and subsequent triggering of deep neuroinflammation processes, e.g., macrophage infiltration as well as microglial and astrocyte overactivation (e.g., cytokine release). The extreme size and proximity of many bomb explosions leads to most victims experiencing all three types of blast-related insults to the head, often despite wearing protective body armor (e.g., goggles and helmet). Thus, we have chosen to combine the first two exposures (i.e., primary and secondary) in our animal model as they are the easiest to stepwise simulate and are highly relevant. Likewise, we have found that the combined insult readily produces an enhanced TBI compared with blast wave or projectile strike (weight drop) alone exposure, having marked pathophysiological and behavioral changes that can manifest both in the acute and chronic phase (Arun et al, 2024). This TBI model, however, can be easily adjusted (i.e., via attenuating the blast wave pressure and weight drop height) within a range of mild to moderate severity that has low mortality and allows for potential full neurological recovery when studying the efficacy of nutritional interventions and drug therapeutics.

Delayed onset of many of the symptoms along with overlap with those of other disorders can lead to diagnosis issues for those affected by TBI (Bramlett and Dietrich, 2015). Thus, clinical and preclinical studies are crucial for assessing the extent of behavioral and molecular changes associated with the diseases to advance studies in this field. Our current study focused on the use of standardized rodent models mimicking human disease state(s) to advance studies in this field. The TBI resulting from simulated blast exposures in these animals is currently being used to mimic blast-related injuries sustained in field-like conditions by soldiers (Rama et al., 2018; Sajja et al., 2018). Blast TBI is the leading cause of combat casualties in which the primary blast overpressure (BOP) waves generated by high intensity explosives lead to diffuse axonal brain injury (Yarnell et al., 2013; Zhou et al., 2018; Bryden et al., 2019). The overpressure energy produced causes concussive damage (e.g., propagated shear forces) to the brain and associated structures within the cranium.

On the other hand, the animal model known as underwater trauma (UWT) exposure is a brief but significant psychological stressor (Richter-Levin, 1998) that produces an acute stress reaction (ASR) and may develop into an acute stress disorder (ASD) or PTSD-like condition if not alleviated or especially repeated. UWT, as predominantly applied when using rats, causes acute and lasting consequences of “anxiety-like” behaviors (e.g., restlessness and weight loss) (Ardi et al., 2014; Ritov and Richter-Levin, 2014). In humans, if symptoms of an ASR or ASD persist for longer than 1 month, individuals can develop PTSD, which presents with an anxiety component characterized by re-experiencing similar sensory input, leading to avoidance and hypervigilance behaviors.

In the second part of our studies, we focused on a nutrition-based approach as a countermeasure for prevention of TBI and PTSD-related debilitations in warfighters. We understand that any trauma to the brain initiates a complex cascade of internal events that can lead to vascular damage, ischemia, excitotoxicity, inflammation, and neuronal cell loss (Lu et al., 2019). Dietary n-3 polyunsaturated fatty acids (PUFAs) are crucial building blocks of neuronal membranes and can be converted to potent anti-inflammatory metabolites (Bazan et al., 2011; Mayurasakorn et al., 2011; Dyall and Michael-Titus, 2008). Nutritional deficiency of n-3 PUFA has been identified as a risk factor for neurocognitive performance and neuropsychiatric disorders. The n-6 and n-3 PUFAs strongly compete in shared biochemical pathways, as based upon their respective ratios and levels in the diet (Lands et al., 1992; Blasbalg et al., 2011).

Previous work suggests that the n−3 polyunsaturated fatty acids α-linolenic acid (ALA), eicosapentaenoic acid (EPA), docosapentaenoic acid (DPA), and docosahexaenoic acid (DHA) are relatively deficient in the present-day Western diet (e.g., from a lack of seafood consumption), including that of the USA, and is instead very high in n-6 PUFAs (e.g., from overconsumption of seed oils and red meat) such as linoleic acid (LA), i.e., 10–30:1 (Blasbalg et al., 2011). This imbalance can lead to adverse consequences as n-3 PUFAs have potent anti-inflammatory, inflammation resolving, and neuroprotective properties, whereas n-6 PUFAs (e.g., arachidonic acid) primarily stimulate inflammation responses. Diet-induced changes in PUFA composition can also modify neuronal cell membrane fluidity and signal transduction (Calder, 2011), and DHA, with the highest degree of polyunsaturation, plays a central role in influencing these neuronal states. All these mechanisms can influence the relative vulnerability or resilience to TBI and traumatic stress. Such diet-derived susceptibilities should be readily and safely correctible through dietary supplements (e.g., ocean fish oil); therefore, the results of this research are highly translatable. Here, we explored the possibility of whether feeding adult rats a n-3 and n-6 PUFA-enriched diet increases their vulnerability to TBI and traumatic stress, in part by influencing the composition of the gut bacterial microbiome.

Additionally, physical and psychological insults to the brain, i.e., TBI and PTSD, can cause the release of HPA-axis-driven stress hormones (e.g., glucocorticoids) that circulate in the bloodstream and then trigger changes to the architecture and function of gut tissues. This in turn leads to loss of the intestinal lumen-epithelium barrier and dysbiosis and leakage of the gut microbiota, which sets off a cascade of immune cell processes (George et al., 2021). Not only does this promote exposure of the brain to elevated circulating inflammation factors (e.g., cytokines) but it also deprives it of important metabolites facilitated by normal gut microbial activity, i.e., nutrient (e.g., short chain fatty acids: acetate, propionate, and butyrate) and neurotransmitter (e.g., dopamine and serotonin) production. Dietary n-3 PUFAs can increase intestinal lumen integrity and likewise decrease gut inflammation (Durkin et al., 2021). Thus, we sought to further identify the intersection as well as unique molecular patterns associated between these two brain insults, with an emphasis on the influence of PUFA nutrition and the gut microbiome.

## 2 Methods

All animal experiments were conducted in accordance with the Animal Welfare Act and other federal statutes and regulations relating to animals and experiments involving animals, and adhered to principles stated in the Guide for the Care and Use of Laboratory Animals (NRC Publication 2011 edition), as performed under an Institutional Animal Care and Use Committee (IACUC)-approved protocol in an AAALAC International accredited facility with a Public Health Services Animal Welfare Assurance. Young adult male 8–9 week-old Sprague Dawley rats that weighed 270–290 g (Charles River Laboratories, Wilmington, MA, USA) were housed at 20–22°C (12 h light/dark cycle) with free access to food and water *ad libitum*.

### 2.1 Subjects and diets

In our study, 72 rats were equally divided into two intervention arms (TBI arm: n = 18 sham, n = 18 TBI exposure. UWT arm: n = 18 sham, n = 18 forced underwater submersion). Groups were equally assigned to one of three diets, with six rats per diet exposure group. Two experimental diets consisted of isocaloric rodent chows, i.e., containing 1% or 8% of energy (en%) as derived from linoleic acid (n-6 PUFA); 1 en% as α-linolenic acid (n-3 PUFA); and no other n-3 PUFAs, which have been used by others to produce in rodents a tissue deficiency of DHA and related physiological disturbances, including brain function (Alvheim et al., 2012). These diets were custom made for the study and followed the National Institutes of Health guidelines for the general nutritional requirements of rodents by using an AIN-93G formulation starting base (modified formula #s: 181161 and 181162, Dytes Inc., Bethlehem, PA, USA). The total protein, carbohydrate, and fat content of both diets is stated by the manufacturer as 18, 46, and 35 en%, respectively. Thus, they are characterized as medium fat diets. A separate group of rats were given a standard animal-facility provided diet (house chow), which is well-balanced in all PUFAs, including EPA and DHA as 0.5 en% combined (Prolab RHM 3000, LabDiet, Purina/PMI Nutrition International, Richmond, IN, USA). The total protein, carbohydrate, and fat content of this diet is stated as 25, 49, and 15 en%, respectively, with 4 and 0.5 en% coming from linoleic and α-linolenic acid, respectively. Thus, the house chow is higher in protein and lower in fat, especially n-6 PUFAs, compared with the other two diets. We confirmed manufacturer-reported fatty acid compositions of the three diets as being accurate within 10% for random food samples (n = 4 each; data not shown), using total lipid extraction, methyl ester derivative, and gas chromatography methods (DeMar et al., 2008). Likewise, preliminary experiments on sham animals verified that moderately prolonged feeding (1–2 months) of the two n-3 PUFA-deficient diets versus house chow (n = 6 each) leads to significant imbalances of n-6 to n-3 PUFA and/or decreases in DHA content (approximately two-fold; p < 0.05) in their livers, a primary organ for the storage of PUFA reserves; however, as reported by others (DeMar et al., 2004), this was protected against within the brains by DHA conservation mechanisms (data not shown).

**Figure 1:**
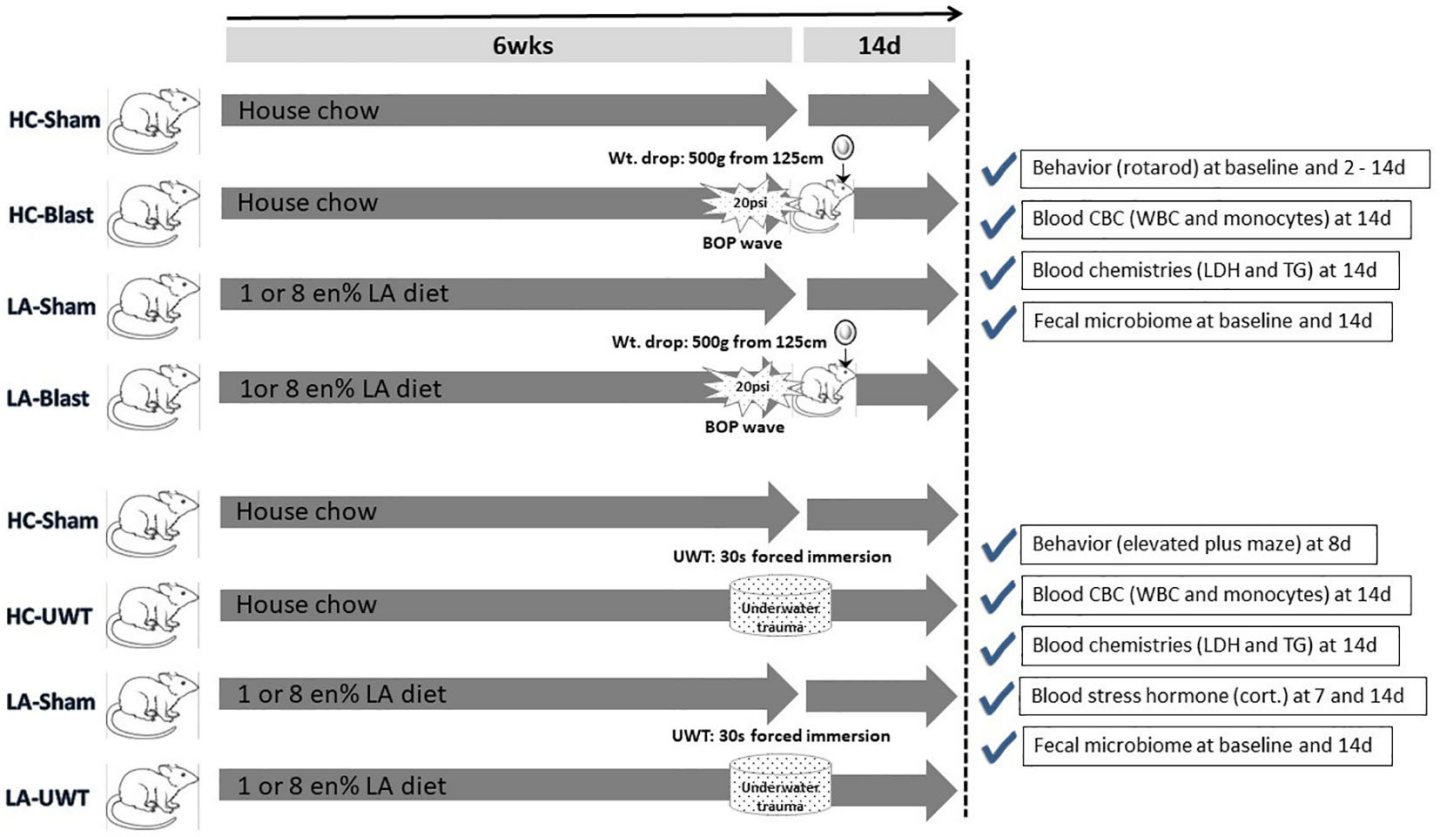
Experimental timeline showing the entire study design. In brief, rats were raised for 6 weeks on one of three diets: house chow, enriched with 1 energy (en)% as linoleic acid (LA), or enriched with 8 en% LA. The latter two diets are thus elevated in n-6 PUFA content (i.e., LA) but contain no long chain n-3 PUFAs, e.g., EPA or DHA. The animals were then exposed to blast-plus-weight-drop-induced TBI (top four arrows) or UWT-induced acute stress (bottom four arrows), along with their respective shams. At baseline and out to 14 d post-insult, the animals were behavior tested and subjected to blood and fecal microbiome assessments, which are further described within the accompanying check marked boxes.

To create military-relevant pathological changes in the brain that can in turn impact gut integrity, the animals are then exposed to high-fidelity simulated blast waves plus a weight drop-induced concussion (Marmarou et al., 1994; Sajja et al., 2018), i.e., a traumatic brain injury (TBI), or an underwater trauma (UWT) psychological stressor (Richter-Levin, 1998; Moore et al., 2012), to be described below in the methods section. Both TBI- and UWT-exposed animals were fed one of the three different diets for 6 weeks prior to insult and continued thereafter: standard house chow (HC) and chows enriched with 1 or 8% of calories/energy as linoleic acid (1 or 8 en% LA diet). The experimental plan is shown as **Figure 1**.

### 2.2 Diet and intervention validation

To broadly validate the effects of diet and exposure on the models, collected tissues were quick-frozen fresh and reserved for future studies on related changes in gene expression, abundances, and activities of proteins (i.e., transcriptomics, proteomics, and metabolomics), as for within the brain, retina, and plasma. This was carried out to identify novel biomarkers of TBI and acute psychological stress, especially under an exacerbating nutritional deficiency of omega-3 fatty acids. In the beginning stages of the planning of this current study, when we were testing the efficiency of the three diets to produce changes in the brain, we subjected the brains of four animals on each diet that were exposed to blast-TBI, along with age-matched shams, to coronal sectioning and colloidal silver staining to detect axonal degeneration by bright field light microscopy.

### 2.3 Exposures

#### 2.3.1 Primary and secondary blast model

A closed head TBI model consisting of blast overpressure (BOP) wave exposure (primary blast injury) coupled with a weight drop concussion (Marmarou method) (secondary blast injury) was used on a group of adult male rats (n=8 each diet). Isoflurane inhalation (2–3%) anesthetized animals were subjected once to BOP (18 psi) inside an Advanced Blast Simulator (ABS) that generates a shock wave following the rapid rupture of a canvas membrane by compressed air. This was immediately followed by dropping a 500 g metal weight from 125 cm above onto a stainless-steel disc affixed to the rat’s skull midway between the lambda and bregma, whereas equivalent diet groups of shams (SH; n=8 each) received handling and anesthesia alone.

#### 2.3.2 Underwater trauma

In parallel, a group of rats were subjected to an UWT stressor model (Richter–Levin method) that consisted of 30 s of swimming and habituation in a 20-gallon tank half-filled with room temperature 0.1% (w/v) saline to help prevent lung inflammation from any aspiration, followed by 30 s of forced whole-body immersion using a perforated plunger. This triggers in the animals the extreme fear response associated with the sensation of near drowning. Shams (SH) received 1 min of free swimming.

#### 2.3.3 Brain histopathology by silver and senescence detector staining

At 14 days following TBI or UWT exposure, animals were deeply anesthetized by 5% isoflurane inhalation for 6 min, and then they were gravity line perfused transcardially with physiological saline to cause blood exsanguination, followed by phosphate-buffered 4% paraformaldehyde saturated with picric acid to fix the tissues. Prior to saline perfusion, blood was taken by cardiac puncture via a syringe and placed in chilled EDTA and heparin vacuum collection tubes. Some blood was centrifuged at 3,000 × g for 10 min to obtain the plasma fraction. Whole blood and plasma were then used for blood work assessments, as to be described. Brains were removed and post-fixed by immersion for up to 6 h in phosphate-buffered 4% paraformaldehyde, and then were switched overnight to buffered 20% sucrose solution followed by one change to fresh sucrose, for cryopreservation purposes. Fixed brains were sectioned, stained, and mounted on microscope slides by submission to FD Neurotechnologies, Inc. (Ellicott City, MD, USA). Brains were cut into serial coronal sections (30–50 μm) at 11 evenly spaced positions (bregma 1.0 mm to –8.3 mm), and mounted in triplicate. These sections cover the entire regions of the cortex, hippocampus, optic tracts, amygdala, thalamus, hypothalamus, mid-brain, and cerebellum. Silver staining, which is highly reactive toward exposed cellular proteins, was performed on the sections derived from the TBI-exposed animals to reveal, as a brown to black color, the presence of any degenerating axonal fiber tracts of the neurons (DeMar et al., 2016). Likewise, floating brain sections from the UWT-exposed animals were provided to us and then slide mounted and stained to reveal as a blue coloration the presence of any prematurely aged neurons, using a senescence detection kit (BioVision, Mipitas, CA, USA) according to the manufacturer’s instructions (Arun et al., 2020). Senescence detector staining is highly reactive toward aging neurons, which overexpress the enzyme β-galactosidase, but not to presenescent, quiescent, or immortal cells. For all prepared slides, mosaic (12 × 14) pictures of the different brain regions of interest were taken using an Olympus BX61 microscope (Olympus Corporation, Center Valley, PA, USA) and the Stereo Investigator virtual image tool (MBF Biosciences, Williston, VT, USA). The intensity of the silver or sequence staining in the captured images was quantified using densitometry (lumens/mm2), as assessed by Image-Pro Premier software (Media Cybernetics Inc., Rockville, MD, USA). For the densitometry measurements, those cells that stained intensely were included and those cells that showed <50% of maximum staining intensity were excluded. A total of four shams and four TBI or four UWT-exposed animals were evaluated for each diet, i.e., house chow, 1 en% LA, and 8 en% LA; however, three, two, and three animals, respectively, were used for the corresponding shams of the TBI-exposed group and their values combined as expected neutral controls for background signal determination. Group comparisons of the averaged staining densities were carried out for sham versus TBI- or UWT-exposed animals using a Kruskal–Wallis one way analysis with a *post-hoc* Dunn’s test, and between the diets alone using ANOVA.

### 2.4 Behavioral, blood, and microbiome assessments

#### 2.4.1 Rotarod

Rats were pretrained for 2 days on a rotarod device (Rotamex-5; Columbus Instruments, Columbus, OH, USA), on which they had to walk on a rotating spindle set at 10 rpm for 45 s. Animals were then tested at 5–35 rpm over 60 s at baseline and then at 2–14 days post-TBI. Latency to fall is reported. This tests for brain impairments (e.g., motor cortex) in balance and coordination.

#### 2.4.2 Elevated plus maze

Animals were placed in the center of an elevated plus maze (EPM) device (Model #: LE840, Harvard Apparatus/Panlab, Holliston, MA, USA) consisting of a raised platform with double intersecting open and closed arms (i.e., risky and safe) and allowed to freely explore it for 5 min, at 8 days post-UWT. The time spent on the arms is reported. This tests for brain (e.g., amygdala) impairments in composure (fear-control) and exploration.

#### 2.4.3 Blood markers

At 7 and 14 days following UWT exposure only, animals were lightly anesthetized through the brief inhalation of 2–3% isoflurane; a blood sample (approximately 200 µl) was collected by lateral tail vein draw, using a 1cc syringe and 27G butterfly needle catheter, and then placed in ice-chilled microfuge tubes containing sodium heparin as an anticoagulant. The blood was immediately centrifuged to obtain the plasma, which was stored at −80°C. Animals were allowed to recover from anesthesia in their home cages. Alternatively, at 14 days post-TBI and -UWT, animals were deeply anesthetized through the prolonged inhalation of 4–5% isoflurane and then euthanized by terminal blood exsanguination followed by decapitation. Exsanguination was carried out by heart puncture with a 10cc syringe and 18G needle, after surgically opening the thorax. Whole-blood draws (approximately 3 ml) were placed inside tubes (Vacutainer, Becton Dickinson, Franklin Lakes, NJ, USA) containing anticoagulant, i.e., di-potassium (K2) EDTA or sodium heparin, that were then gently hand-mixed and kept chilled on ice.

Corticosterone levels (Cort; ng/ml) of plasma samples from tail vein blood taken at 7- and 14-days post-UWT were determined within the investigator’s lab (i.e., BINT Branch) using a species-specific multiplex immunoassay array (MILLIPLEX MAP Rat Stress Hormone Magnetic Bead Panel, Millipore Sigma, Burlington, MA, USA). After loading the samples and then developing the assay, the arrays were read on a multiplexing imaging platform (Luminex MAGPIX–xPONENT^®^; Bio-Techne/R&D Systems, Minneapolis, MN, USA), which calculates the results from a standard curve. In brief, the instrument scans for the specific binding of tagged (e.g., fluorescent nano-bead) antibodies to register and quantify the corticosterone.

Terminal whole-blood samples taken at 14 days post-TBI and -UWT were submitted on the same day to the WRAIR Clinical Pathology Department for a chemistry panel and complete blood count assessment, with processing undertaken within 24 h post-collection. Chemistries (heparin-treated samples) for lactate dehydrogenase (LDH; U/L) and triglyceride (TG; mg/dl) levels were performed on plasma, as isolated by blood centrifugation, using a clinical chemistry analzyer (VITROS 350 System; Ortho-Clinical Diagnostics/Quidel Ortho, San Diego, CA, USA). In brief, the instrument uses colorimetric reactions to quantify the two analytes. Likewise, complete blood counts (ETDA-treated samples) for levels (density per µl) of total white blood cells (WBC) and monocyte fraction were performed on whole blood, using a clinical hematology analyzer (XT-2000iV; Sysmex America, Lincolnshire, IL, USA). In brief, the instrument uses fluorescent dye-based flow cytometry to identify and count the immune cell populations.

#### 2.4.4 Fecal microbiome

Fecal pellets were collected directly from the animals in the morning 1–2 days before and 14 days after blast plus weight drop or underwater trauma exposure, by holding the subject over a new absorbent under pad until a fresh stool sample (1–2 droppings) was deposited on it, which was immediately transferred with tweezers to a cryotube and then quick frozen on dry ice and stored at −80°C. Massaging of the animals was used to promote defecation. A fresh area of the under pad was used each time and the tweezers were cleaned with isopropyl alcohol wipes between animals. Microbial DNA was isolated from the fecal pellets using a bacterial nucleic acid extraction kit (QIAamp PowerFecal Pro DNA, Qiagen, Germantown, MD, USA). The quality and quantity of the prepared DNA was checked by UV spectrometry (NanoDrop 8000, Thermo Fisher Scientific), dye fluorometry (Qubit 4, Invitrogen/Thermo Fisher Scientific), and capillary gel-electrophoresis (2200 TapeStation, Agilent Technologies, Santa Clara, CA, USA). The DNA was fragmented and then analyzed on a metagenomic sequencing machine (MiSeq, Illumina, San Diego, CA, USA) using primers for the V3 and V4 regions of the prokaryote-ubiquitous 16S rRNA gene, which are variable according to the bacterial type and absent in the mammalian host cells, for amplification (Karl et al., 2023). In brief, amplified DNA was sequenced, producing 300 bp paired-end reads in a single run. Pairs were merged with q2-vsearch, were quality checked, had chimeras removed, and were de-multiplexed using q2-demux and DADA2 (Callahan et al., 2016), using QIIME2 v.2020.82 (Bolyen et al., 2019). Amplicon sequence variants (ASV) were aligned with MAFFT (Katoh and Standley, 2013) via q2‐alignment and used to construct a phylogeny with FastTree2 via q2-phylogeny (Price et al., 2010). To assign taxonomy, a naïve Bayes Classifier was trained on the 16S rRNA V3–V4 region with the specific primers and the Greengenes v13.8 (McDonald et al., 2012) 99% operational taxonomic unit database of reference sequences using the q2-feature-classifier via Classify-Sklearn.

### 2.5 Data analysis

#### 2.5.1 Physiologic outcomes

Outcome measures were sampled after either sham or experimental exposure. Outcomes were analyzed within each experiment using linear modeling of continuous diet compositions testing *Outcome* ~ [*ALA*] + [*LA*] + *Condition*. Tables were formatted with *stargazer* (Hlavac, 2022). Categorical comparisons using t-tests were made within diets comparing sham vs. treatment, and between diets comparing treatment-exposed models. Power analysis was performed for each significant outcome using Cohen’s D to quantify effect size with alpha = 0.05, n = 36 using *pwr* (Champely, 2020), and *effsize* packages in R (Torchiano, 2020).

#### 2.5.2 Preparation

The 16S OTU tables were prepared in QIIME. Relative abundance tables were exported into R for downstream analysis. Features observed in fewer than half of the samples were removed, and data were not interpreted below the family level as OTUs at this classification level could not be further classified. Relative abundance data are available in **Appendix A**.

#### 2.5.3 Alpha diversity

Alpha diversity is defined as reflecting the number or relative abundances of bacteria taxa within the averaged samples. Shannon-, Chao1-, and Simpson-based alpha diversities were calculated using QIIME and exported into R for analysis and visualization. The effects of diet were evaluated using linear modeling of *diversity metric ~* [*ALA*] + [*LA*] + *Time* + *Treatment exposed* to characterize the continuous effects of dietary components on outcome. Diet group differences were compared within and across diets using a t-test and ANOVA. Within diets, alpha diversity was compared before and after exposure. Across diets, treatment (not sham)-exposed models were compared against other diet alpha diversity.

#### 2.5.4 Beta diversity

Beta diversity is defined as reflecting the variability in identities of bacteria taxa among the individual samples. Beta diversity was calculated in R using the *vegan* package (Oksanen et al., 2022). Distance matrices were produced from relative abundance tables at the family level. Bray distances were calculated using relative abundance and Jaccard distances were calculated using a binary-transformed dataset. All beta diversity comparisons were performed using an ADONIS test, which under brain insult conditions (TBI vs. UWT) showed a significant distance between experiments; therefore, each experiment was independently analyzed. Within experiment analysis was performed by testing *Distance* ~ [*ALA*] + [*LA*] + *Time* + *Treatment exposed* with both Jaccard and Bray distances.

#### 2.5.5 Composition

Effects of experimental treatments on fecal microbiome composition were evaluated across the measured taxonomic levels using linear modeling of *scale* (*Abundance*) ~ [*ALA*] + [*LA*] + *Time* + *Treatment exposed*. Microbiome abundances were centered and scaled to reduce the effect of non-normal feature distribution on modeling and to enable interpretation of the results between features with different abundances. Categorical comparisons were made within and between diet groups following the same strategy as outlined in alpha diversity above; however, Kruskal–Wallis was chosen due to the non-normal distribution of the microbiome abundances.

#### 2.5.6 Microbiome feature selection

The process described in 2.4.1 identified outcomes that were significantly affected by either experimental exposure or dietary status. Outcomes with models that had at least a moderate effect size (>0.2 for adjusted R2) were used to build stepwise microbiome models. Exhaustive feature selection is a method that rationally identifies predictors that balance model fit with error rate to conclude what combination of possible predictors best explains a target outcome, balancing both positive and negative effects on the target (Chowdhury and Turin, 2020). Stepwise selection was limited to the same number of predictors (3) as in the diet + exposure models. The *leaps* package was used to perform exhaustive predictor selection (Miller, 2020). Once predictors were identified, they were centered and scaled to aid in interpretation given the wide difference in the magnitude of relative abundance between predictors. The final model was run using stepwise predictors using the base R package lm() function. The distribution of residuals was checked for homoscedasticity using *lmtest* (Achim and Torsten, 2002). All outcome measure models were homoscedastic.

## 3 Results

### 3.1 Diet and intervention validation

We observed a significant presence of degenerated axons in the blast-TBI exposed animals above shams, especially in the white matter rich optic tracts, with the highest tendency in those fed the omega-3-deficient diet containing 1 en% of LA. There were, however, no significant differences found between the three diets, and thus, it was only a verification that the model was producing a robust brain injury (**Supplementary Figure S1**). As for the UWT-exposed animals, it is known that this model mainly produces HPA-axis-driven neurochemical/hormonal imbalances in the brain that at an acute exposure stage do not result in readily observed physical changes to the neuronal cells; however, there are reports of microglia overactivation in other animal models of acute and especially chronic psychological stress (Enomoto and Kato, 2021). Likewise, we tried looking at brain sections obtained from UWT-exposed pilot animals on each diet (n = 6) with a highly sensitive immuno-stain for detecting senescent/aged neurons (i.e., β-galactosidase expression), but did not find any significant increases over shams of dysfunctional cells within the hippocampus and amygdala in the primary fear memory centers. Independent of UWT exposure, interestingly, there was across the board a significantly greater number of prematurely aging neurons in both omega-3-deficient diets (**Supplementary Figure S2**).

Power analysis described in 2.5.1 for UWT (**Appendix B**) and TBI models (**Appendix C**) found that future studies could be improved by larger sample sizes. UWT model power was calculated as 0.052 for 7-d corticosterone, 0.63 for 14-d corticosterone, 0.48 for LDH, 0.53 in open arm EPM, and 0.31 in closed arm EPM. TBI models were powered at 0.59 for LDH, 0.75 for 6 d rotarod, and 0.38 for 14-d rotarod.

### 3.2 UWT physiologic effects

Linear modeling the effects of UWT using linear regression of *outcome measure* ~ [*LA*] + [*ALA*] + *condition* found that these predictors explained only 10–30% of the outcome measures (**Table 1**). Given that blood samples were only collected in the post-exposure, condition time was not included as a factor. With this approach, all measured outcomes were found to produce statistically significant models, although only LDH, time in the EPM closed arm, and 14-d corticosterone had a moderate effect size. Across outcomes, [ALA] had a greater effect size than [LA]. Notably, [ALA] had greater negative effects on the EPM closed arm and LDH. UWT, at 14 d post-exposure, was not found to cause significant neuronal cell perturbations in brain regions, i.e., the hippocampus and amygdala, controlling the formation of fear-memory-related behaviors (e.g., elevated plus maze impairments), as assessed by histopathology using senescence detection staining.

**Table 1:** LM results of UWT outcomes.

UWT outcome measures								
	WBC***	Monocyte***	LDH***	TG***	Open***	Closed***	Cort 7d***	Cort 14d***
LA	40.59	14.24^***^	-129.11^***^	-8.28^*^	1.30	-17.05^***^	10,487.75^**^	12,269.80^***^
	(36.38)	**(2.99)**	**(16.97)**	**(3.30)**	(1.19)	**(2.02)**	**(3,267.85)**	**(2,773.15)**
ALA	364.67	238.87^***^	-2,380.89^***^	-117.08	33.25	-340.90^***^	230,474.10^***^	283,320.40^***^
	(674.32)	**(55.49)**	**(314.51)**	(61.25)	(21.97)	**(37.45)**	**(60,328.32)**	**(51,401.74)**
UWT	683.54^***^	-13.02	-244.93^***^	**-17.05**	-16.11^***^	19.11^*^	3,479.03	-52,173.16^***^
	**(143.42)**	(11.80)	**(66.89)**	**(13.03)**	**(4.67)**	**(7.97)**	(13,197.91)	**(10,932.20)**
Constant	3,565.78	-1,120.24^***^	15,135.35^***^	855.16^*^	**-126.90**	2,083.04^***^	-1,051,995.00^**^	-1,324,956.00^***^
	(3,914.10)	**(322.11)**	**(1,825.58)**	**(355.55)**	**(127.50)**	**(217.39)**	**(350,498.40)**	**(298,360.10)**
Observations	245	245	245	245	245	245	231	245
R^2^	0.13	0.11	0.23	0.08	0.08	0.30	0.11	0.29
Adjusted R^2^	0.12	0.10	0.22	0.07	0.07	0.29	0.10	0.28
Residual Std. Error	1,120.95	92.25	522.83	101.82	36.51	62.26	100,157.20	85,446.79
F Statistic	12.48^***^	10.28^***^	24.40^***^	7.42^***^	6.90^***^	33.69^***^	9.20^***^	32.40^***^

^*^ *p*<0.05; ^**^ *p*<0.01; ^***^ *p*<0.001.

Bolded values highlight significant results corresponding to p-values <0.05.

**Figure 2:**
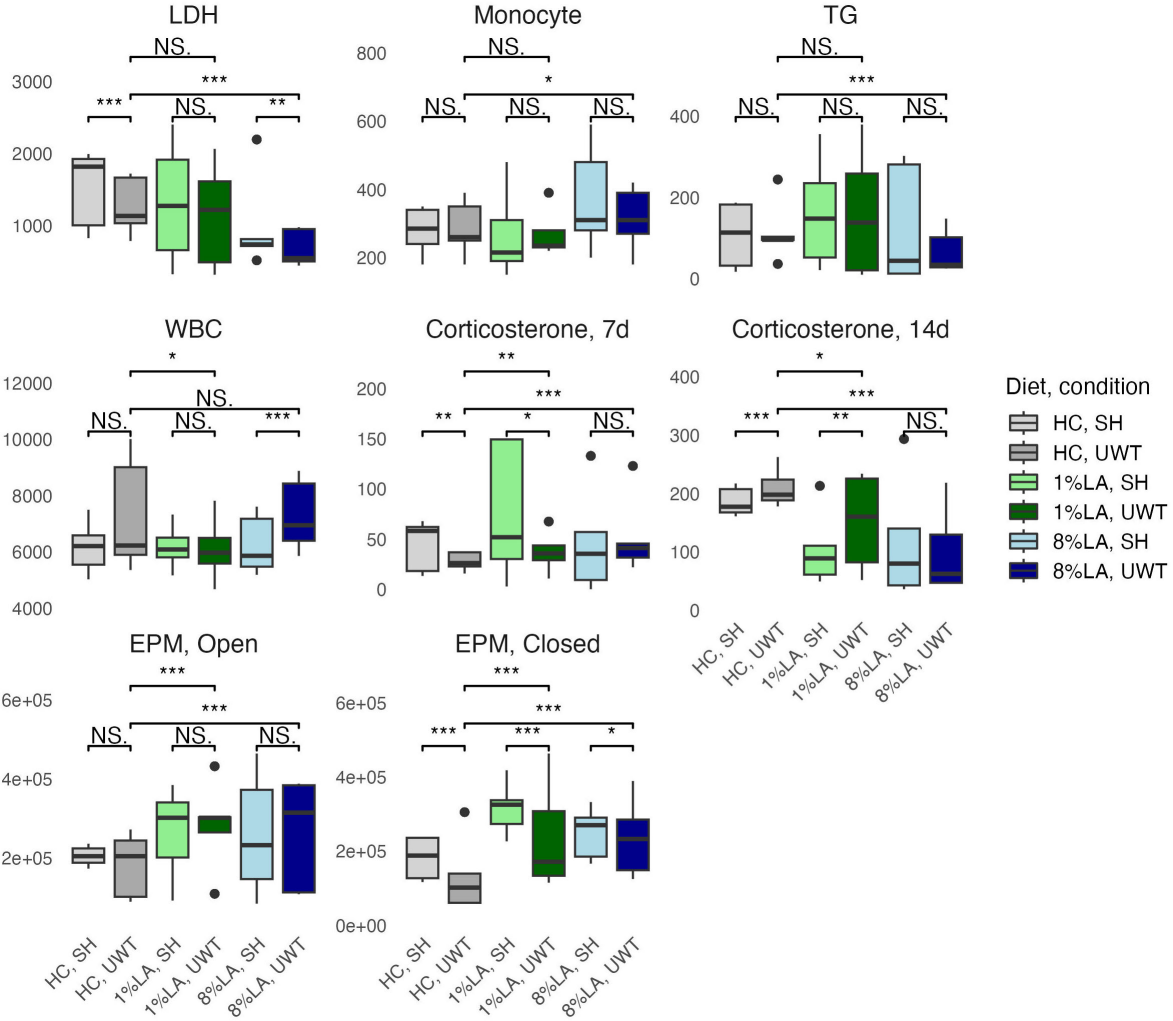
Physiologic outcomes of UWT exposure comparing shams with matched UWT-exposed models using a t-test (n=6 per group). LDH, lactate dehydrogenase; TG, triglyceride; WBC, white blood cell; EPM, elevated plus maze; HC, house chow, 1%LA, 1%en LA diet; 8%LA; 8%en LA diet; SH, Sham; UWT, underwater test. NS = *p*>0.05, *p<0.05, **p<0.01, ***p<0.001.

Using a t-test to evaluate categorical within-diet differences between sham and UWT, we found that WBC count, LDH, TGs, and corticosterone 14-d post exposure were significantly different between UWT and sham (**Figure 2**). WBC count was significantly higher in UWT (HC), whereas LDH was significantly reduced in the UWT group (HC and 8% LA). Corticosterone was significantly reduced in UWT models at 7 and 14 d within the HC and 1% LA diets (**Figure 2**).

Across diets, behavioral testing shows that across UWT-exposed diet groups, both LA-enriched diets spent significantly more time in both elevated plus maze (EPM) open and closed arms than the HC diet. Within diets, when comparing sham and UWT, there was a significant decrease in the time spent in EPM closed arms within the UWT-exposed model in every diet. Linear modeling found that these categorical observations of across-diet differences were most attributable to [ALA], not [LA] in the diet.

### 3.3 TBI physiologic effects

Linear models of TBI outcomes found that [LA] and [ALA] had significant positive effects on monocyte and TG levels, but a negative effect on LDH. For these outcomes, [ALA] showed a greater magnitude of effect (**Table 2**). TBI exposure significantly increased monocytes and LDH and reduced TG and day 6 rotarod behaviors of balance and coordination. [ALA] had greater effect sizes than [LA] in these TBI models. Of the outcomes that were measured in the TBI and UWT models, effect magnitudes were nearly identical. Adjusted R2 values found that only LDH and TGs had moderate effect sizes.

**Table 2:** LM results of TBI outcomes.

TBI outcome measures						
	WBC	Monocyte***	LDH***	TG***	Rotarod, 6d*	Rotarod, 14d**
LA	-38.27	9.51^*^	-101.34^***^	17.18^***^	0.31	-0.42
	(56.33)	**(3.95)**	**(12.24)**	**(2.38)**	(0.40)	(0.43)
ALA	-975.95	205.84^**^	-2,207.95^***^	364.43^***^	5.00	-11.59
	(1,049.67)	**(73.65)**	**(228.05)**	**(44.27)**	(7.46)	(7.92)
TBI	364.40	50.51^**^	314.75^***^	-28.15^**^	-4.34^**^	-1.96
	(222.28)	**(15.60)**	**(48.29)**	**(9.38)**	**(1.58)**	(1.68)
Constant	11,395.81	-859.77^*^	13,381.75^***^	-1,888.32^***^	**2.16**	98.17^*^
	(6,085.68)	**(426.98)**	**(1,322.17)**	**(256.69)**	**(43.23)**	**(45.94)**
Observations	238	238	238	238	238	238
R^2^	0.02	0.10	0.46	0.32	0.04	0.05
Adjusted R^2^	0.01	0.09	0.45	0.31	0.02	0.04
Residual Std. Error	1,704.47	119.59	370.31	71.89	12.11	12.87
F Statistic	1.90	8.85	66.16	36.52	2.90	4.48

^*^ *p*<0.05; ^**^ *p*<0.01; ^***^ *p*<0.001.

Bolded values highlight significant results corresponding to p-values <0.05.

**Figure 3:**
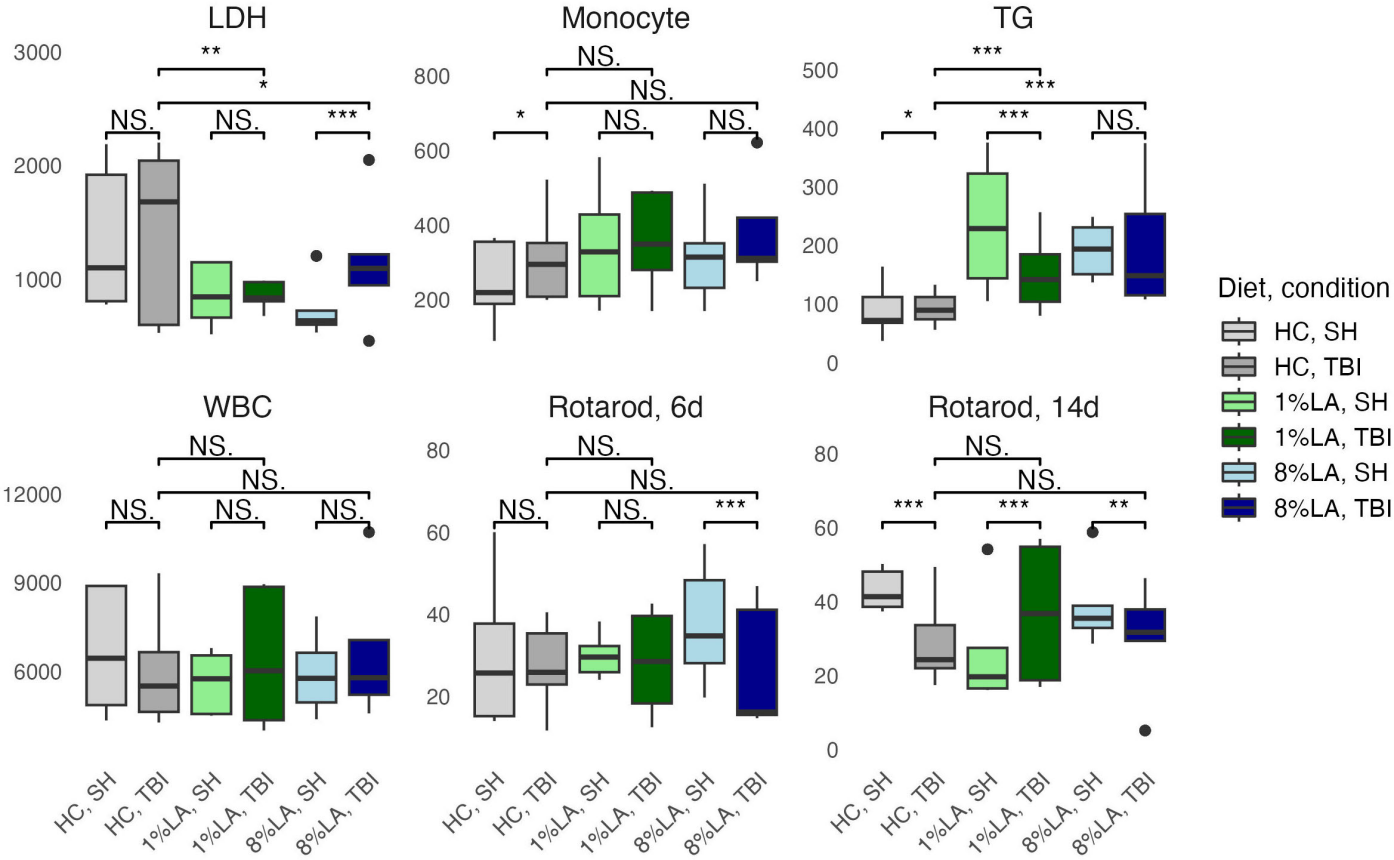
Physiologic outcomes of TBI exposure comparing shams with matched TBI-exposed models using a t-test (n=6 per group). LDH, lactate dehydrogenase; TG, triglyceride; WBC, white blood cell, HC, house chow, 1%LA, 1%en LA diet; 8%LA, 8%en LA diet; SH, Sham; TBI, traumatic brain injury. NS = *p*>0.05, *p<0.05, **p<0.01, ***p<0.001.

A t-test comparison of TBI and sham groups within diets found that TBI had significantly higher levels of monocytes (HC), LDH (8% LA), and TGs (HC) but lower levels of TGs in the 1% LA diet (**Figure 3**). Rotarod testing at day 6 showed no between diet differences and that TBI had significantly lower balance times in the 8% LA only. At day 14, balance times were significantly lower in the TBI group within the HC and 8% LA but were higher in the 1% LA.

Between diets comparisons in TBI models found that terminal LDH was significantly lower in both LA-enriched diets compared with HC. Terminal TGs were elevated in LA-enriched diets. Rotarod latency to fall times were not significantly different between diets.

### 3.4 Microbiome alpha diversity

Simpson diversity was significantly reduced by dietary [LA] and [ALA] in the UWT models (**Table 3**). Treatment exposure was not significant for either model. Only time was significant for the Shannon diversity of the UWT models. The UWT and TBI models had a small to moderate effect size.

**Table 3:** Effects of diet and condition on Shannon and Simpson alpha diversity.

	Shannon		Simpson	
	TBI*	UWT*	TBI***	UWT***
LA	-0.06	-0.05	-0.01	-0.06^**^
	(0.08)	(0.13)	(0.02)	**(0.02)**
ALA	-1.94	-1.21	-0.51	-1.39^***^
	(1.56)	(2.33)	(0.42)	**(0.33)**
Time	0.37	-2.20^**^	-0.07	-0.05
	(0.43)	**(0.64)**	(0.12)	(0.09)
Treatment Exposed	0.25	0.33	0.10	-0.01
	(0.39)	(0.57)	(0.10)	(0.08)
Constant	22.26^*^	21.27	6.48^*^	11.71^***^
	**(9.09)**	(13.56)	**(2.46)**	**(1.95)**
Observations	48	48	48	48
R^2^	0.25	0.24	0.39	0.47
Adjusted R^2^	0.18	0.17	0.33	0.42
F Statistic (df = 4; 43)	3.60^*^	3.45^*^	6.76^***^	9.37^***^

^*^ *p*<0.05; ^**^ *p*<0.01; ^***^ *p*<0.001.

Bolded values highlight significant results corresponding to p-values <0.05.

**Figure 4:**
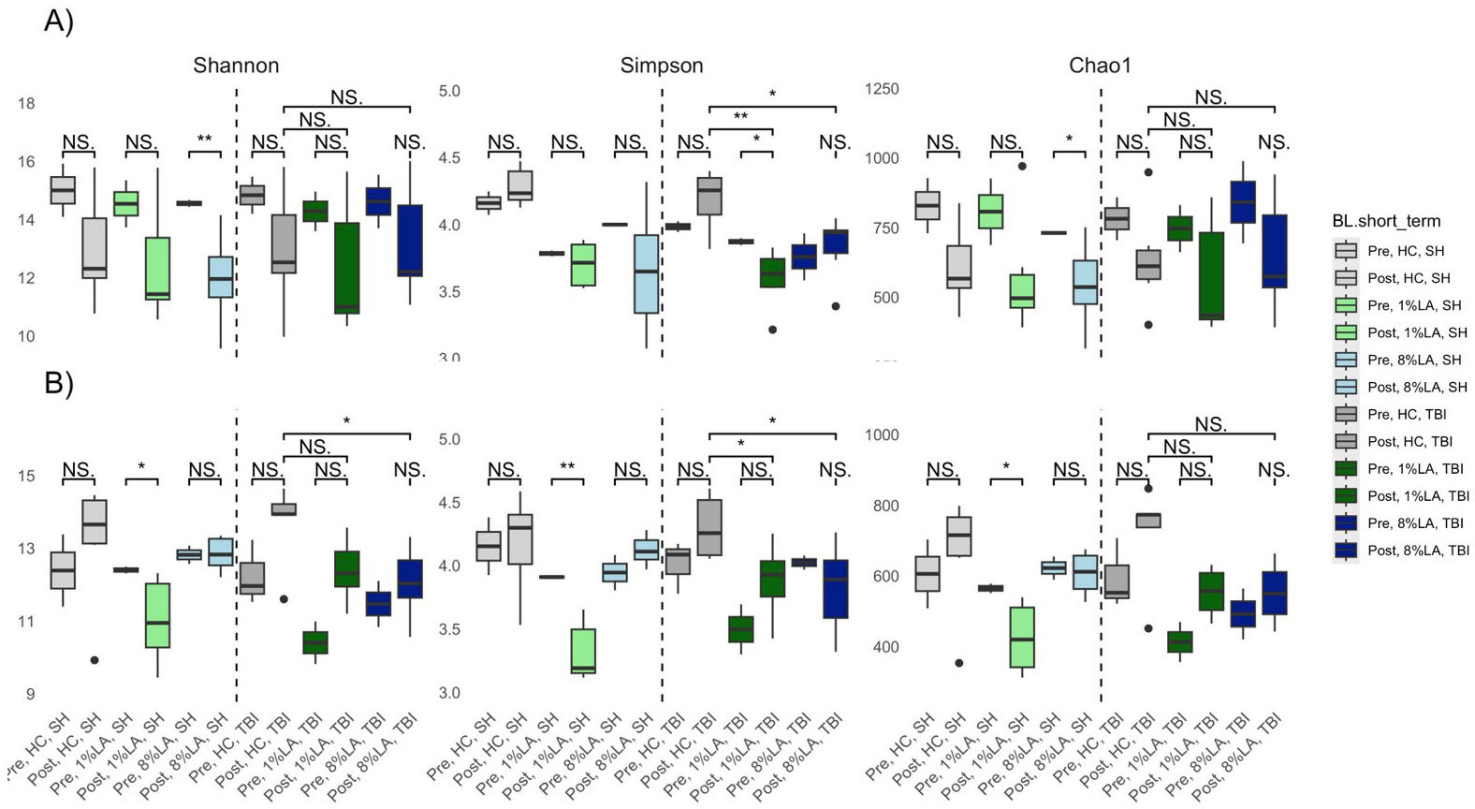
Effect of diet and brain insult exposure on three measures of alpha diversity (Shannon, Simpson and Chao1) of UWT **(A)** and TBI **(B)** models using a t-test (n=6 per group). HC, house chow; 1%LA, 1%en LA diet; 8%LA, 8%en LA diet, SH, sham; UWT, underwater test; TBI, traumatic brain injury. NS = *p*>0.05, *p<0.05, **p<0.01, ***p<0.001.

Categorically, within TBI diets, alpha diversity was significantly decreased by sham exposure in the 1% LA models (**Figure 4**). Within the UWT group, alpha diversity was significantly decreased by sham exposure in the 8% LA group and by UWT exposure in the 1% LA UWT group. Alpha diversity was only disrupted in LA-supplemented models.

### 3.5 Microbiome beta diversity

We found a significant difference between TBI and UWT when comparing all samples in both Bray (ADONIS: p<0.01, R2 = 0.09, Residual = 0.91) and Jaccard (ADONIS: p<0.001, R2 = 0.08, Residual = 0.92); therefore, analysis proceeded within TBI and within UWT models.

**Figure 5:**
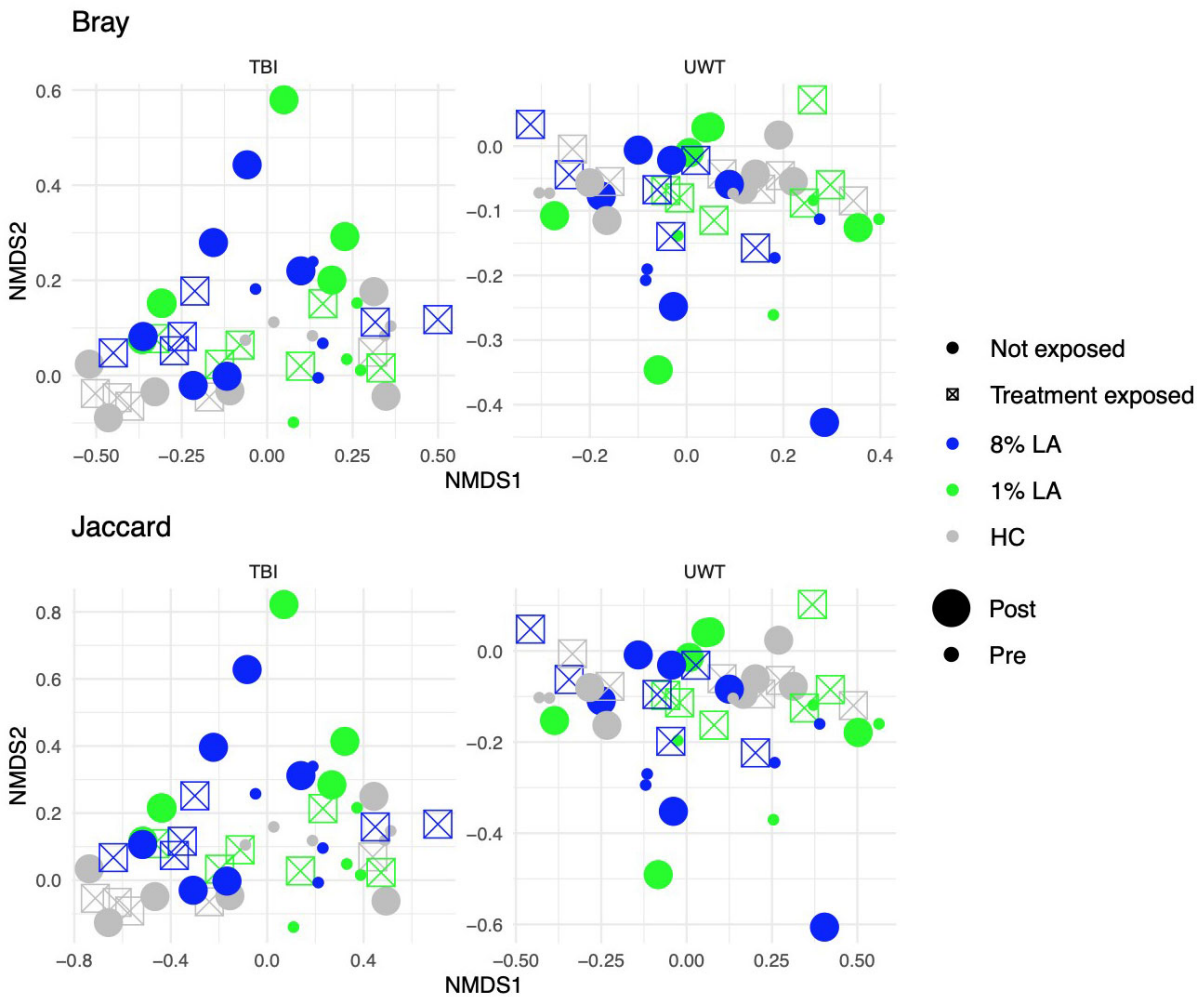
Effect of diet and brain insult exposure on beta diversity within TBI (left panels) and UWT (right panels) models. The top row of panels shows beta diversity measured by Bray–Curtis distances, whereas the bottom row displays beta diversity measured by Jaccard distances. Each panel compares the impact of diet and brain insult on the microbial communities, highlighting differences in community composition (Bray–Curtis) and the presence/absence of taxa (Jaccard).

Diet effects were not observed in the UWT model (**Table 4**) but were present in TBI (**Table 5**). Within-experiment distances were tested with ADONIS controlled against the HC diet, baseline condition, and SH condition by *β* ~ *Diet* + *Time* + *Treatment exposed* (**Figure 5**).

**Table 4:** ADONIS beta diversity results within the UWT model.

Effect	Bray					Jaccard			
	Df	Sum of Sq.	R2	F	P	Sum Of Sq.	R2	F	P
1 % LA	1	0.29	0.06	3.13	***	0.44	0.06	2.91	***
8 % LA	1	0.27	0.06	2.88	**	0.42	0.05	2.73	***
**Time**	**1**	**0.18**	**0.04**	**2.00**	*	**0.29**	**0.04**	**1.92**	*
Tx Exposure	1	0.06	0.01	0.6	NS	0.1	0.01	0.63	NS
*Residual*	*43*			*NA*	*NA*			*NA*	*NA*

*p*<0.01; *p<0.05; **p<0.01; ***p<0.001.

NA, Not Applicable; NS, Not Significant.

Bolded values highlight significant results corresponding to p-values <0.05.

**Table 5:** ADONIS beta diversity results within the TBI model.

Effect	Bray					Jaccard			
	Df	Sum of Sq.	R2	F	P	Sum Of Sq.	R2	F	P
1 % LA	1	0.12	0.02	1.04	NS	0.21	0.02	1.15	NS
8 % LA	1	0.18	0.03	1.55	NS	0.30	0.03	1.62	.
**Time**	**1**	**0.56**	**0.09**	**4.80**	***	**0.79**	**0.08**	**4.30**	***
Tx Exposure	1	0.11	0.02	0.90	NS	0.16	0.02	0.90	NS
*Residual*	*43*	*5.05*	*0.84*	*NA*	*NA*	*7.88*	*0.84*	*NA*	*NA*

*p*<0.01; *p<0.05; **p<0.01; ***p<0.001.

NA, Not Applicable; NS, Not Significant. Bold values highlight significant findings (p < 0.05).

Within the TBI model, only time was significant (Bray ADONIS, P<0.01, R2 = 0.09, Residuals = 0.8; Jaccard P<0.001, R2 = 0.8, Residuals = 0.84). The 8% LA diet was nearly significant (Jaccard ADONIS, P=0.08, R2 = 0.03, Residuals = 0.84). TBI exposure was not significant (**Table 5**).

In contrast, within the UWT model (**Table 4**), diet was highly significant for 8% LA (Bray ADONIS, P<0.01, R2 = 0.06, Residuals = 0.83) and 1% LA (Bray ADONIS, P=0.01, R2 = 0.06). Brain insult exposure by either model did not have a significant effect on microbiome beta diversity.

### 3.6 Compositional effects of diet and time

#### 3.6.1 Phyla level

**Figure 6:**
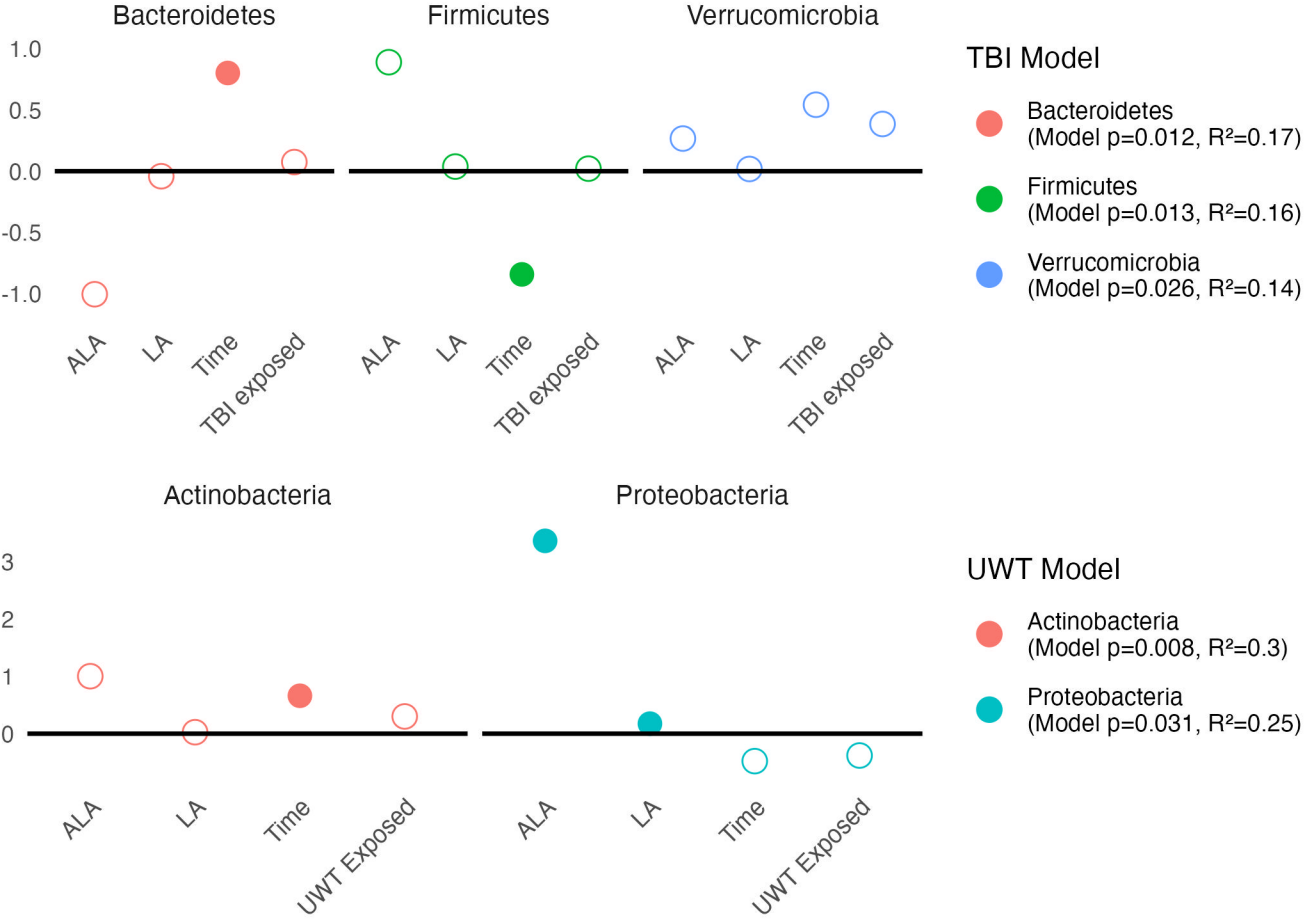
Estimates of phyla-level linear models of *scale(abundance) ~ [ALA]+[LA]+Time+Treatment exposed_yes/no_*. Estimate values of the TBI (top panel) and UWT (bottom panel) models are shown. Open circles represent non-significant predictors. Filled circles identify significant model predictors. Model R2 and *p*-values are shown in the legend. ALA, alpha linolenic acid; LA, linoleic acid; TBI, traumatic brain Injury; UWT, underwater test.

Linear modeling found that there were different effects of the LA-enriched diets between experimental groups. At the phyla level, [ALA] and [LA] only affected *Proteobacteria* abundance in the UWT model, in which [ALA] had a larger effect size than [LA]. Exposure was not significant for any phyla (**Figure 6**).

Pre-post Kruskal–Wallis within TBI groups found *Proteobacteria* (1% LA) and *Bacteroidetes* (HC) were significant increased by TBI exposure, whereas *Firmicutes* (HC) were significantly decreased. These findings were not observed in the sham models. Within the UWT model, there were significant increases in *Actinobacteria* (1% LA), *Firmicutes* (HC), and *Tenericutes* (HC), and a decrease in *Bacteroidetes* (HC). These effects were also not observed in the sham controls.

#### 3.6.2 Class level

**Figure 7:**
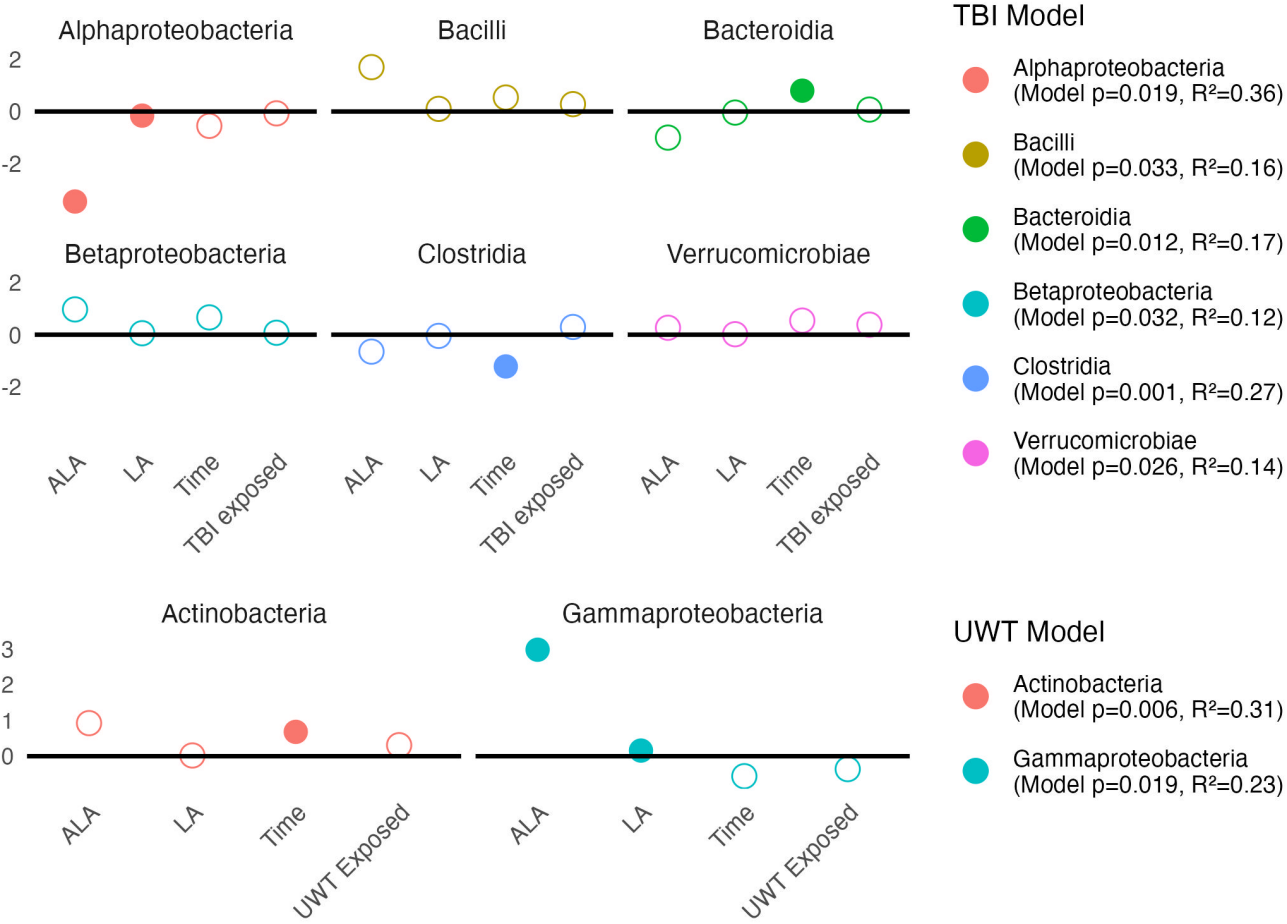
Estimates of class-level linear model *scale(abundance) ~ [ALA]+[LA]+Time+Treatment exposed_yes/no_*. Estimate values of the TBI (top panel) and UWT (bottom panel) models are shown. Open circles represent non-significant predictors. Filled circles identify significant model predictors. Model R2 and *p*-values are shown in the legend. ALA, alpha linolenic acid; LA, linoleic acid; TBI, traumatic brain injury; UWT, underwater test.

Linear modeling found that [ALA] and [LA] had a significant effect on two *Proteobacteria* subclasses. Within the TBI model (**Figure 7**, top panel), *Alphaproteobacteria* was significantly decreased by [ALA], with a small effect observed by [LA]. Within the UWT model (**Figure 7**, bottom panel), *Gammaproteobacteria* was significantly increased by [ALA], while [LA] had a much smaller effect size (**Figure 7**).

A Kruskal–Wallis test within diets and between timepoints showed that class level changes within the TBI model were more likely to occur in the [LA] (n-6 PUFA)-enriched diets, whereas the opposite was observed in the UWT model. Within the TBI model, there were significant increases in *Bacilli* (1% LA), *Bacteroidia* (HC), and *Betaproteobacteria* (HC and 1% LA), and decreases in *Clostridia* (1% LA and 8% LA) and *Erysipelotrichi* (HC). Only one of these effects *(Clostridia)* was also shared with its matched sham control. Within the UWT models, there was an exposure-related increase in *Actinobacteria* (8% LA), *Clostridia* (HC), and *Mollicutes* (HC), and a decrease in *Bacteroidales* (HC).

#### 3.6.3 Order level

**Figure 8:**
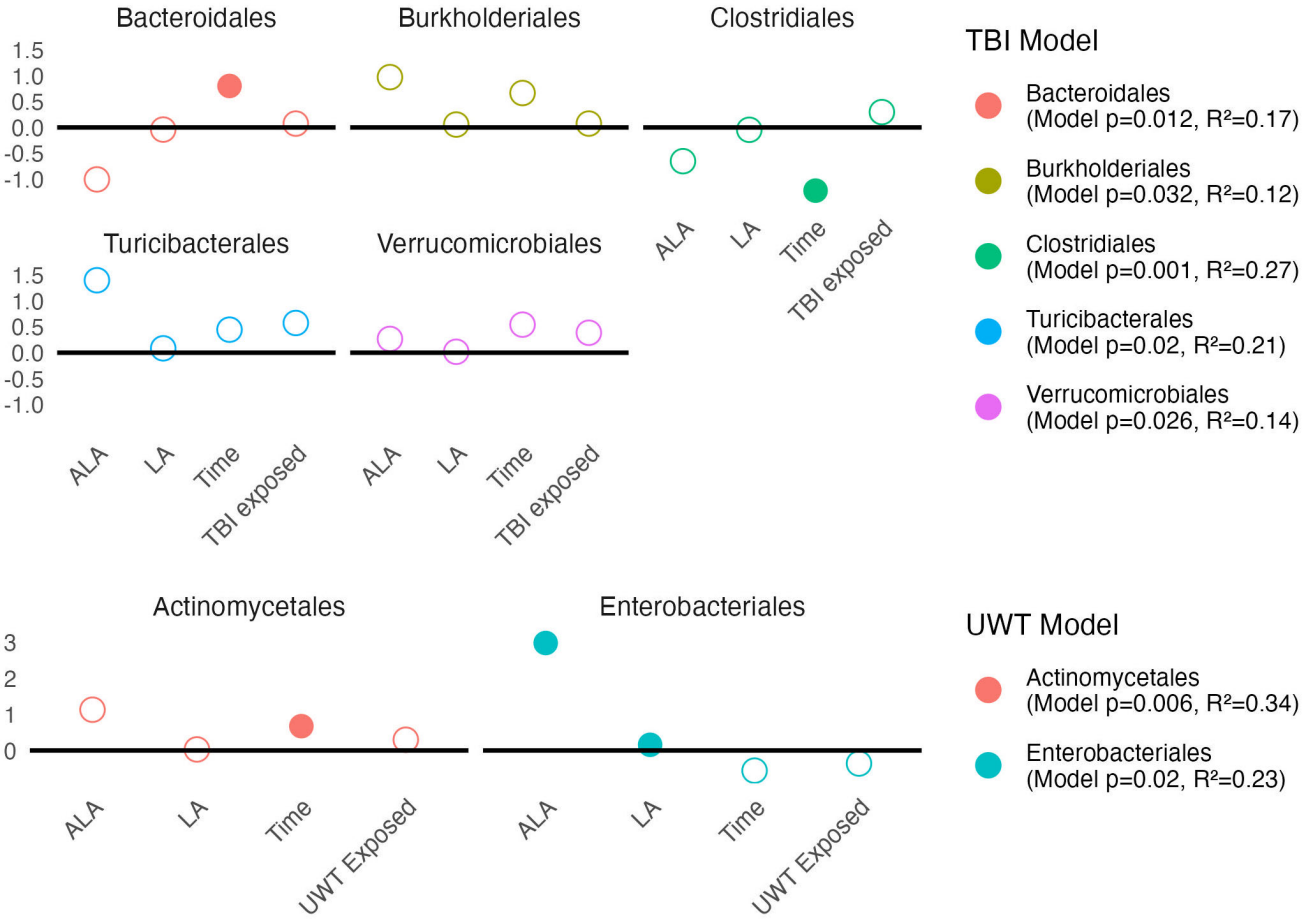
Estimates of order-level linear model *scale(abundance) ~ [ALA]+[LA]+Time+Treatment exposed_yes/no_*. Estimate values of the TBI (top panel) and UWT (bottom panel) models are shown. Open circles represent non-significant predictors. Filled circles identify significant model predictors. Model R2 and *p*-values are shown in the legend. ALA, alpha linolenic acid; LA, linoleic acid; TBI, traumatic brain injury; UWT, underwater test.

Modeling showed that at the order level, [ALA] and [LA] only had a significant effect on *Enterobacteriales* abundance. TBI or UWT exposure was not significant for any order (**Figure 8**).

Observed changes in *Alphaproteobacteria* were attributable to the RF32 order in the UWT and TBI models. TBI-associated increases in *Betaproteobacteria* were attributable to *Burkholderiales*, which was found to significantly increase post-TBI in the 1% LA and was nearly significant in the 8% LA diet (p=0.07), but not for the HC. The *Bacilli* TBI effect was not significant for any sub-assignment of that class, although this trend appeared to be split between a non-significant post-exposure increase in *Lactobacillales* and *Turicibacterales*. Furthermore, within the TBI diets, there were significant pre-post increases in *Bacteroidales* (HC) and *RF32* (8% LA, nearly significant in 1% LA p=0.07), and decreases in *Clostridiales* (1% LA and 8% LA), *Erysipelotrichales* (HC), and *RF39* (1% LA). Within UWT diets, there were significant increases in *Actinomycetales* (1% LA), *Clostridiales* (HC), and *RF39*, (HC) and decreases in *Bacteroidales* (HC). Notably, *Verrucomicrobiales* was nearly significantly increased in both sham (p=0.053) and UWT (p=0.07) HC groups.

### 3.7 Effect of microbiome composition on outcome measures

#### 3.7.1 Phyla level

At the phyla level, *Actinobacteria* exerted a negative effect on the time spent in the EPM closed arms and corticosterone levels at day 14 (**Table 6**). *Verrucomicrobia* reduced LDH levels within the UWT model and in time spent in the EPM closed arms. *Bacteroidetes* were associated with an increase in LDH in the TBI model. *Tenericutes* were associated with significantly reduced 14-d corticosterone levels. *Cyanobacteria* was associated with increased LDH only in the TBI model. Between the UWT and TBI models, different predictors were selected between the same outcomes. Effect sizes on the microbiome were small to moderate, and R2 values were like the diet + condition models.

**Table 6:** Stepwise feature selection of microbiome predictors of outcome measures.

	UWT			TBI	
	LDH *	EPM, closed ***	Cort. 14d (10^-3^) ***	LDH ***	TG NS
*Firmicutes*	-16.44 (10.52)			18.16 (18.85	19.07 (15.37)
*Actinobacteria*		-28.47** **(11.01)**	-41.52** **(16.09)**		-23.72 (14.24)
*Verrucomicrobia*	-24.74** **(9.93)**	-24.12** **(10.71)**			
*Bacteroidetes*			24.39 (14.80)	37.92** **(17.99)**	
*Tenericutes*	11.83 (10.25)		-62.90*** **(15.70)**		
*Cyanobacteria*		14.39 (10.79)		20.47** **(7.73)**	-17.87 (15.35)
Constant	115.11*** **(9.46)**	140.73*** **(10.21)**	227.21*** **(14.20)**	105.95*** **(6.78)**	158.33*** **(13.81)**
Observations	35	35	35	36	36
R2	0.21	0.40	0.38	0.36	0.09
Residual Std. Error	55.98	60.42	84.00	40.66	82.88
F Statistic	2.72	6.96	6.33	7.46	2.13`

*p<0.05, **p<0.01, ***p<0.001.

Outcome measures affected by experimental interventions were evaluated for UWT (left) and TBI (right) to observe the effects of bacterial phyla on outcomes. The selection was limited to a maximum of three predictors, mirroring the number of predictors incorporated in the diet (three levels) and condition outcome models. Predictors were centered and scaled so that the effects of taxa with different abundances could be better compared. Bold values highlight significant findings (p < 0.05).

## 4 Discussion

### 4.1 Physiologic outcomes

Analysis of WBC, monocyte, LDH, and TG blood levels across experiments found that diet but not treatment exposure had a small but significant effect on outcome measures. Of the regression models testing experimental variables that reached significance, the effect sizes of diet and exposure were small to moderate. The three experimental diets showed higher sensitivity to disruptions in WBCs, LDH, corticosterone elevation, and rotarod behavioral impairments in balance and coordination. These findings across both experiments suggest that n-6 PUFA enrichment may increase susceptibility to cellular and immunologic dysfunction independent of brain insult exposure. Within the UWT model, the greatest behavioral effect of dietary n-6 PUFA enrichment was increased time spent in closed arms of the elevated plus maze. This suggests that fear, or in essence, anxiety-like behaviors, as noted in these animals, may be especially sensitive to this dietary feature. Biologically, corticosterone at 14 d post-exposure was also significantly affected by dietary n-6 PUFA intake. The longitudinal changes in cortisol, the analog of corticosterone that is present in humans, is complex in PTSD. Classically, immediately after the initial trauma in human subjects, cortisol spikes and falls over time; the degree of hypocortisolemia may be associated with symptom severity and PTSD development (Heim et al., 2000). A review of the evidence emphasizes that the development of a hypocortisol state following traumatic exposure may be an important factor in at least some PTSD biotypes (Meewisse et al., 2007). The corticotropin-releasing hormone receptors (CRH-Rs), which regulate cortisol and corticosterone production, are highly susceptible to sensitization. Typically, a strong CRH negative feedback signal will *de-sensitize* the receptor; however, if certain circumstances are met, the receptor can instead become sensitized to the presence of cortisol (or corticosterone), which leads to chronically low basal levels of cortisol in individuals suffering from trauma disorders. This has been suggested as a factor in how childhood trauma causes persistent phenotypic changes (Toth et al., 2015), and further studies have shown that CRH-Rs variants may be associated with susceptibility to trauma disorders (White et al., 2013). Our finding that n-6 PUFA experimental diets significantly reduced day 14 corticosterone levels may be clinically important because it identifies a potential modifiable dietary factor that contributes to hypocortisolemia, a PTSD risk factor. We hypothesize that n-6 PUFA is a dietary factor that might increase the risk of developing PTSD, potentially through a cortisol or CRH-R sensitizing mechanism. As measured values of 7-d corticosterone were significantly lower in the UWT-exposed models than in the sham swim rats, this hypothesis could be further explored. Higher frequency sampling may be important in the post-trauma exposure to capture post-traumatic corticosterone increases as this may have resolved within 7 d post-exposure.

TBI and trauma occur unpredictably with variable time from event to medical care (Mohamadpour et al., 2019). It has been difficult to develop acute phase post-injury interventions that have been effective in blunting the biologic cascade that occurs after these exposures (Bramlett and Dietrich, 2015). Previous work has identified that the acute period is of extreme biological importance in determining the severity of long-term injury (Prins et al., 2013). Delay from exposure to point of care may reduce the efficacy of post-exposure treatments given the speed of the biologic response immediately after exposure. Populations with a high incidence of TBI and psychologic trauma (especially the military) may benefit most from prophylactic interventions, in which even those with small effect sizes could have a high population level benefit.

Owing to the relatively small group sizes (n = 8) used in this study, it was difficult to consistently detect behavioral deficits in the animals on the three diets under the two brain insult conditions, i.e., blast-TBI and UWT. Likewise, the TBI model used in the study is mild to moderate in intensity and its duration of effect. Thus, it is not surprising that significant behavioral deficits on the rotarod were absent 6 d post-exposure. Typically, our other behavioral studies require at least 16 animals per group to overcome the variances in individual animal intrinsic-performance capabilities on the rotarod. Likewise, in a separate study that used 10 animals, we found at 2 d post-TBI that house-chow-diet-fed animals showed a 30% decrease (p < 0.05) in latency times on the rotarod, which resolved by day 6. Thus, we could try reanalyzing the rotarod data at 2 days out and/or as normalized to the animal’s baseline performance scores. The elevated plus maze testing for the UWT-exposed animals was also likely underpowered, leading to an inability to detect behavioral deficits in increased exaggerated fear response (“anxiety”) as characterized by avoidance of the high-risk environment represented by the open arms. In other studies, however, we have noted that animals under acute psychological stress more readily show signs of increased exploratory hyperactivity on all arms of the elevated plus maze (i.e., distance traveled), which in this case with the small powering may have led to an overall tendency to spend less time in the closed arms. The non-concurrent increase in open arm time can be partially explained by the animals frequently hesitating to move while in the central zone of the maze (i.e., the intersection of the arms), which was not counted as part of the time scoring. To help eliminate some of these issues, which reflect the complexity of even occupancy exchanges between the arms of the maze, the data are often processed by other investigators (Mazor et al., 2009) using the following “anxiety” index formula: 1 − [((open arm time/300-s) + (open arm entries/total entries))/2]. Unfortunately, in our study, we only recorded arm time and not the number of arm entry events.

### 4.2 Alpha diversity

Alpha diversity disruption was only observed in experimental diets across both studies. This suggests that the presence of dietary n-6 PUFA may negatively affect microbiome diversity. These effects were observed in both sham- and brain-insult-exposed experimental diet groups, suggesting that despite the 6-week period of diet acclimation before the intervention, there was still a temporal effect as a confounding variable. Previous research has suggested that the post-TBI microbiome state contributes to the development of TBI sequelae (Simon et al., 2020). Little research has been carried out associating alpha diversity with trauma or TBI outcomes. Premorbid n-6 PUFA dietary status should be further evaluated as a risk factor for developing trauma and TBI-related disorders.

### 4.3 Beta diversity

The TBI and UWT groups were analyzed independently. There was a large degree of residual variability when accounting for all interventions. Across experiments, the 8% LA diet and time showed the greatest effect on beta diversity with condition being non-significant in both experiment’s ADONIS. This suggests that between group differences reached significance because of both sham and experimental exposure, but neither experimental condition itself significantly affected beta distances. High levels of n-6 PUFA intake, observed in the 8% LA diet, exerted significant and nearly significant effects on the UWT and TBI exposures, respectively. This suggests that a high n-6 PUFA diet supported a microbiome that was compositionally distinct from standard house chow, with a better balance of n-3 to 6 n-6 PUFA. Cumulatively, these beta diversity effects show that there was significant variance in microbiome composition between the measured groups, especially between diets.

### 4.4 Composition and effects on physiologic outcomes

There were a host of TBI-, UWT-, and diet-related effects. The main observed compositional changes were *Bacteroidetes* and *Proteobacteria*, which were both decreased by UWT exposure. In the TBI group, *Proteobacteria* increased over time. Other studies of traumatic exposures have found prominent increases in *Proteobacteria* and *Firmicutes* post-TBI (Nicholson et al., 2019). Studies evaluating how phyla abundance affects TBI outcomes suggest that some clinically relevant observations, such as lesion size, may be significantly predicted by microbiome composition. *Proteobacteria* abundance has been suggested to be positive correlated with magnetic resonance imaging lesion size in TBI patients, whereas *Firmicutes* has been shown to be inversely correlated using single-feature phyla level linear models (Nicholson et al., 2019). This further suggests that the observation of n-6 PUFA enrichment potentially exposing models to *Proteobacteria* upregulation may be biologically relevant and attenuating increases in *Proteobacteria* changes post-TBI could have the potential to improve outcomes.

Increases in bacteria taxa with a n-6 PUFA-enriched diet suggests that premorbid n-6 PUFA intake may contribute to this change. Likewise, as microbiome suppression by antibiotics has been found to ameliorate TBI sequelae, when given in the subacute period (Uckun et al., 2015), increases in taxa prevalence (possibly *Proteobacteria)* may be more likely to contribute to TBI sequelae than taxa that are decreased by TBI, although this hypothesis needs more evidence.

Deeper classification analysis attributed *Proteobacteria*’s enhancement to *Betaproteobacteria* in the TBI-exposure model across diets. The biological relevance of the *Betaproteobacteria* includes sulfur oxidation into sulfate and the acidification of its environment via hydrogen ion production (Watanabe et al., 2019), as well as environmental nitrification, with studies finding associations between *Betaproteobacteria* population levels and ammonia and phosphorous concentrations with R2 >90% in environmental samples (Martínez-García et al., 2022). It is unknown how TBI affects neuronal cell ammonia levels; however, evidence from other organ systems shows that disrupting circulating ammonia elimination has a strong association with brain concentrations and, thus, the extent of cerebral edema, seizure development, and encephalopathic symptoms (Sharma et al., 2024). Although this is a highly interpretive conclusion, it strongly identifies *Betaproteobacteria* as a potential candidate for contributing to TBI sequelae. This may be worth further research toward a better understanding of the relationship between blood ammonia levels and TBI explicitly.

Within the UWT model, exposure significantly increased *Alphaproteobacteria* and *Actinobacteria* by linear modeling. *Alphaproteobacteria* has an unclear ecological function, especially as we were unable to further subclassify this class given the limitations of only carrying out 16S rRNA analysis and not looking at other characterizing bacterial genes. Although *Proteobacteria* subgroups were somewhat classified with conserved ecological roles, that was not the case with *Actinobacteria.* This phylum has been suggested as a potent modulator of secondary metabolites almost acting as a regulatory promoter of the metagenome (Hoskisson and Fernández-Martínez, 2018); therefore, extrapolations about its potential role in our model microbiomes cannot be reasonably made.

The resulting models included the same number of variables from our initial evaluation of how diet (which was treated as three degrees of n-6 to n-3 PUFA balance) and brain insult condition influenced outcomes. The notable difference in models was that variability in microbiome composition had similar or higher R2 values than the effects of diet and brain condition alone. As feature selection allows the model to find the most suitable variables, it is unknown whether results represent a biologic relevance or overfitting.

However, features identified by this method are largely consistent with previous research. We want to highlight the associations found with *Verrucomicrobia* and reducing both LDH and time spent in the elevated closed arm. *Verrucomicrobia* is a mucin-dependent anaerobe that thrives on mucosa membranes. Previous studies have shown that *Verrucomicrobia* is inversely correlated with the severity of metabolic disease, lipopolysaccharide translocation into circulation, and inflammation status (Derrien et al., 2004). Across studies, *Verrucomicrobia* has presented itself as a candidate microbiome feature for which upregulation could improve host resilience. It has been shown to have immune modulatory effects and is associated with increased IL-10 expression in the gut, which is a cytokine known to markedly suppress inflammation processes (Lindenberg et al., 2019). Given the consistent finding of the benefit of this phyla, future interventional research is warranted regarding specifically upregulating this target. Culture for this microbe is challenging given its specific mucin and anaerobic requirements; therefore, prebiotics, such as the glucose-absorption-reducing drug metformin (Pavlo et al., 2023), may be more effective in increasing *Verrucomicrobia* abundance.

*Tenericutes* (subclassified as *RF39* and *RF32)* were shown to have a strong association with reduced corticosterone levels at 14 d in this study. *Tenericutes* typically exist at low levels in the microbiome but exert an outsized ecological role. Biologically, *Tenericutes* are butyrate producers (Vital et al., 2014), with a high representation of acetate-producing genes but lacking the somewhat common ability of other microbial species to synthesize the mucosal irritant hydrogen peroxide from glycerol (Wang et al., 2020). *RF39* and *RF32* are also notable for their gene content encoding cardiolipin production (Wang et al., 2020), a metabolite (di-phosphatidylglycerol lipid) that has been shown to enhance oxidative processes within the mitochondria, especially under glutamatergic stress (Głombik et al., 2021). Given the large effect size of *Tenericutes* on corticosterone levels, further investigation into its effect on known metabolites, such as cardiolipin, is warranted. Unsurprisingly, there was little overlap in the microbiome features associated with the TBI and UWT models. The UWT model was designed to induce neuroendocrine dysfunction, whereas the TBI model was designed to induce physical axonal and inflammatory injury in the brain. Taken collectively, these results illustrate that in two military-relevant models n-3 to n-6 PUFA intake may affect (even if only to a small magnitude) traumatic sequelae.

Finally, the application of stepwise feature selection to relate microbiome composition with outcome measures may also play an important role in understanding which collection of taxonomies in combination best explains a phenotype. A key limitation of our study was that a testing dataset was not available for validation. Future microbiome studies by others in rats using alternative established traumatic stressor (e.g., inescapable foot shocks) or closed head TBI (e.g., lateral fluid percussion injury) methods may clarify our results by testing our stepwise models against their own. Another limitation of this work was the small sample size per group (n=6) and evaluation of only male models, as females often show sex-specific microbiome responses. Repetition of this study would likely yield different taxonomic changes due to baseline compositional variance between cohorts, especially as our results were produced from a single cohort. However, we would anticipate similar findings that dietary PUFA significantly affects both alpha and beta diversity. Likewise, the blast-TBI and acute traumatic stress exposures would have been translationally better to that occurring in humans if they were instead performed in a ferret or mini-pig model, which also possess a structurally higher-ordered brain, i.e., gyrencephalic (folded cerebral cortex), with better behavioral capabilities; and thus, might also have more similar systemic communication effects on the microbiome. It is also known that the rat gut microbiome composition does not mirror the human equivalent well, whereas mice are considered a better rodent model for these types of studies (Nagpal et al., 2018). Thus, an interesting extension of our work would be to stay with rats as a model, as opposed to using less neurologically advanced mice, but to oral dose some of them prior to the two brain insults with probiotic bacterial strains that are known to reside and maintain gut health in humans.

We are currently in pursuit of two projects that are utilizing the tissues that were collected from the same animals used in this study. The first project is looking at the transcriptomic (gene expression) changes that occurred in the whole retinas, as an indicator of neurosensory (visual system) impairment, and has found that numerous proteins and associated signaling pathways specific to retinal neurons, e.g., photoreceptor cells and endocannabinoid signaling, were significantly altered by both the blast-TBI and UWT exposures, with the highest degree of inhibition found in the animals on the omega-3-deficient diet containing 8 en% of LA. Likewise, we are now pursuing transcriptomic analysis of the whole brain and plasma samples that were collected from these same animals to look at the central nervous system and systemic changes. Overall, it is our desire to eventually perform correlation analysis between the changes found in the gut microbiome and the transcriptome in brain, retina, and/or plasma, as a means of identifying biomarkers (proteins and bacteria) that tightly associate with the occurrence of the blast-TBI and UWT exposures and severity of the behavioral impairments. We also want to support these data in the future by conducting total proteomic and metabolomic assessments of these tissues, using LC/MS-MS methods, as a means of identifying changes in the abundances of the expressed proteins and their enzymatic products (e.g., energy metabolism), including site-specific amino acid shifts in post-translation modifications that can greatly modulate the protein’s function and ability to cause pathological states, e.g., phosphorylation patterns.

This project identified that premorbid dietary composition has a potential role in influencing hypocortisolemia, a risk factor for developing PTSD. To date, there are only limited methods to influence cortisol levels that could reasonably be used in a military deployment. Reducing dietary n-6 PUFA may be a promising method. The gut microbiome appeared to be significantly disrupted by dietary status more so than exposure to TBI or UWT, highlighting that diet has a profound effect on modulating microbiome signaling. Thus further work needs to be undertaken in identifying microbiome features associated with positive outcomes of TBI and PTSD.

## Data Availability

Normalized data files have been uploaded as **Supplementary material**. The raw data supporting the conclusions of this article will be made available by the authors, without undue reservation.

## Author disclaimer

Material has been reviewed by the Walter Reed Army Institute of Research. There is no objection to its presentation and/or publication. The opinions or assertions contained herein are the private views of the author, and are not to be construed as official, or as reflecting true views of the US Department of the Army or the US Department of Defense. Research was conducted under an IACUC-approved animal use protocol in an AAALAC International - accredited facility with a Public Health Services Animal Welfare Assurance and in compliance with the Animal Welfare Act and other federal statutes and regulations relating to laboratory animals.
