# Supplementary material for: The effect of dietary omega-6 fatty acid enrichment in rodent models of military-relevant acute traumatic psychological stress and traumatic brain injury
Source: Front Microbiomes. 2024 Sep 11;3:1430340. doi: 10.3389/frmbi.2024.1430340 (PMC12993493; doi:10.3389/frmbi.2024.1430340)
Supplement: Supplementary file 1 [file DataSheet1.zip › Appendix B.PDF]

Outcome	Cohen’s D	Alpha	Study sample n	Power
LDH	0.32	0.05	36	0.48
Corticosterone 7d	0.02	0.05	36	0.052
Corticosterone 14d	0.38	0.05	36	0.63
EPM, Closed	0.24	0.05	36	0.31
EPM, Open	0.34	0.05	36	0.53

Appendix B – Power analysis of UWT data
