# Supplementary material for: The effect of dietary omega-6 fatty acid enrichment in rodent models of military-relevant acute traumatic psychological stress and traumatic brain injury
Source: Front Microbiomes. 2024 Sep 11;3:1430340. doi: 10.3389/frmbi.2024.1430340 (PMC12993493; doi:10.3389/frmbi.2024.1430340)
Supplement: Supplementary file 1 [file DataSheet1.zip › Appendix C.PDF]

Outcome	Cohen’s D	Alpha	Study sample n	Power
LDH	0.36	0.05	36	0.59
Rotarod 6d	0.44	0.05	36	0.75
Rotarod 14d	0.27	0.05	36	0.38

Appendix C – Power analysis of TBI data
