# Supplementary material for: The effect of dietary omega-6 fatty acid enrichment in rodent models of military-relevant acute traumatic psychological stress and traumatic brain injury
Source: Front Microbiomes. 2024 Sep 11;3:1430340. doi: 10.3389/frmbi.2024.1430340 (PMC12993493; doi:10.3389/frmbi.2024.1430340)
Supplement: Supplementary file 1 [file DataSheet1.zip › Figure Captions.docx]

**Supplemental Figure S1:** A) Representative microscope images of senescence detector-stained brain regions revealing presence of aged neurons for the hippocampus and amygdala (red arrows) of sham and UWT exposed rats, maintained on a 1 en% LA diet.  B) Histopathology outcomes of UWT exposure comparing shams with matched UWT exposed models using ANOVA test between diets (n=4 per group).  *#* *p<0.05*.
 
**Supplemental Figure S2:** A) Representative microscope images of silver-stained brain regions revealing presence of axonal fiber tract degeneration for the optic tract (red arrows) of sham and TBI exposed rats, maintained on a 1 en% LA diet.  B) Histopathology outcomes of TBI exposure comparing combined shams with matched TBI exposed models using Kruskal-Wallis One Way Analysis with post-hoc Dunn’s test in the case of shams (n=3, 2, and 3 from each respective diet) versus TBI (n=4) alone, **p<0.05, **p<0.01, ***p<0.001*; and by ANOVA test between diets, except for shams. *# p<0.05.*
