# Supplementary figures and images for: The effect of dietary omega-6 fatty acid enrichment in rodent models of military-relevant acute traumatic psychological stress and traumatic brain injury
Source: Front Microbiomes. 2024 Sep 11;3:1430340. doi: 10.3389/frmbi.2024.1430340 (PMC12993493; doi:10.3389/frmbi.2024.1430340)

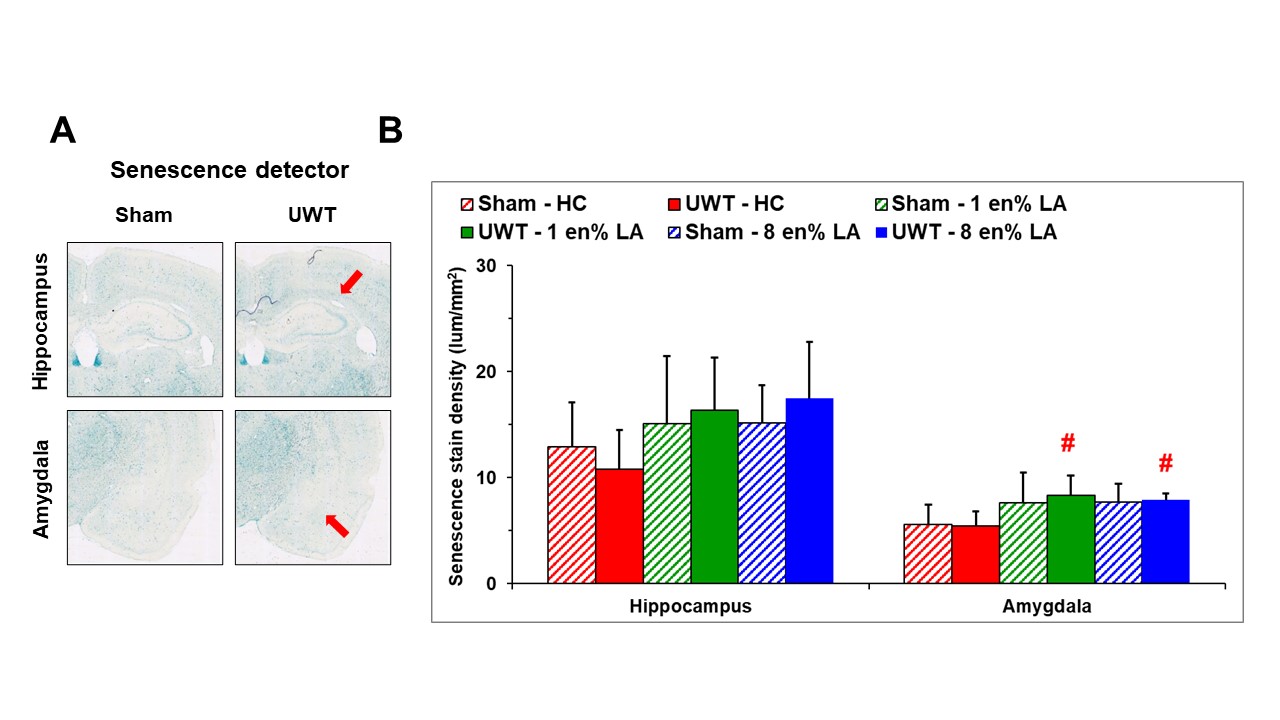

Supplement: Supplementary file 1 [file DataSheet1.zip › Figure S1.JPEG]

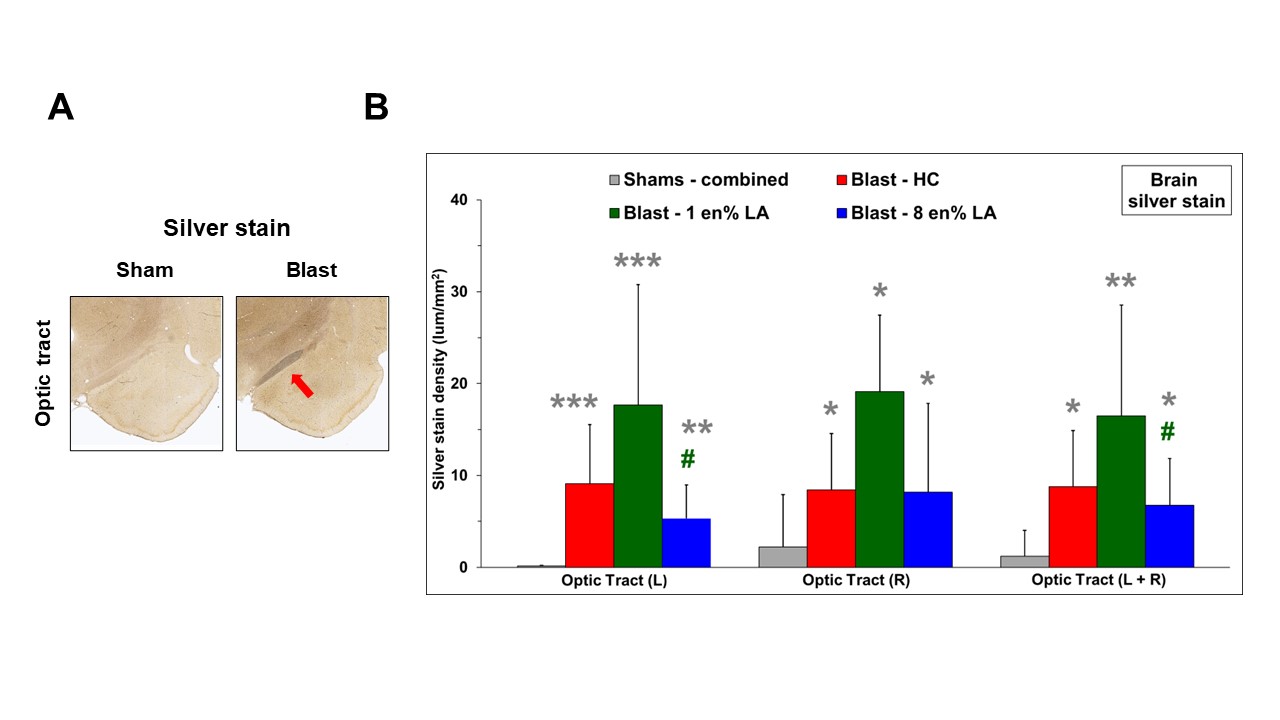

Supplement: Supplementary file 1 [file DataSheet1.zip › Figure S2.JPEG]
